# Comparison of published guidelines for management of coagulopathy and thrombosis in critically ill patients with COVID 19: implications for clinical practice and future investigations

**DOI:** 10.1186/s13054-020-03273-y

**Published:** 2020-09-16

**Authors:** Adam Flaczyk, Rachel P. Rosovsky, Clay T. Reed, Brittany K. Bankhead-Kendall, Edward A. Bittner, Marvin G. Chang

**Affiliations:** 1 Division of Critical Care, Department of Anesthesia, Critical Care and Pain Medicine, Massachusetts General Hospital, Boston, USA; 2 Division of Hematology and Oncology, Massachusetts General Hospital, 55 Fruit Street, Boston, MA USA; 3 Division of Hematology and Oncology, National Institutes of Health, Bethesda, MD USA; 4 Department of Surgery, Division of Trauma, Emergency Surgery, and Surgical Critical Care, Massachusetts General Hospital, Boston, MA USA; 5 Division of Cardiac Anesthesia and Critical Care, Department of Anesthesia, Critical Care and Pain Medicine, Massachusetts General Hospital, Boston, USA

**Keywords:** COVID-19, Coronavirus, Thrombosis, Anticoagulation, Venous thromboembolism, Hematologic monitoring, Therapeutic anticoagulation

## Abstract

Critically ill patients with COVID-19 are at increased risk for thrombotic complications which has led to an intense debate surrounding their anticoagulation management. In the absence of data from randomized controlled clinical trials, a number of consensus guidelines and recommendations have been published to facilitate clinical decision-making on this issue. However, substantive differences exist between these guidelines which can be difficult for clinicians. This review briefly summarizes the major societal guidelines and compares their similarities and differences. A common theme in all of the recommendations is to take an individualized approach to patient management and a call for prospective randomized clinical trials to address important anticoagulation issues in this population.

## Introduction

COVID-19-associated coagulopathy [1] is well established in COVID-19 patients and is marked by a state of profound inflammation with endothelial dysfunction, abnormal flow dynamics, platelet activation, and hypercoagulability resulting in higher rates of thrombotic complications such as deep venous thrombosis (DVT), pulmonary embolism (PE), and microvasculature thrombosis [2–5]. Given the evidence that COVID-19 patients are at increased risk for thrombotic complications, a number of consensus guidelines and recommendations are now available to facilitate clinical decision-making. However, these guidelines are limited by their lack of data from randomized controlled clinical trials. This review provides an overview of major societal guidelines and recommendations that focus on prevention, monitoring, and treatment of COVID-19-associated coagulopathy (CAC). In addition, the similarities and differences between the guidelines are highlighted, and their implications for practice are explored. Lastly, proposed topics for future consideration and study are offered.

## Current societal recommendations: similarities and differences

We review seven major societal recommendations and guidelines addressing the management of coagulopathy in COVID-19 patients: the Centers for Disease Control and Prevention (CDC) [6], International Society on Thrombosis and Haemostasis interim guidance (ISTH-IG) [7], American Society of Hematology (ASH) [8, 9], American College of Chest Physicians (ACCP) [10], Scientific and Standardization Committee of ISTH (SCC-ISTH) [11], Anticoagulation Forum (ACF) [12], and American College of Cardiology (ACC) [13]. These recommendations and guidelines were specifically selected for review because of their focus on anticoagulation in critically ill patients. Moreover, each sponsoring organization has prior expertise in developing evidence-based guidelines and providing health-related recommendations in the arena of venous thromboembolism (VTE). The aims of the major societal recommendations and guidelines vary. The ISTH interim guidance is unique in that it provides guidance on the risk stratification for COVID-19 patients on admission and the management of coagulopathy based on laboratory parameters. Alternatively, ACF, ASH, CDC, ACCP, ACC, and SCC-ISTH offer guidance on in-hospital and post-discharge VTE prevention, treatment, and monitoring.

Tables 1, 2, 3, 4, 5, 6, and 7 highlights six major societal recommendations and guidelines on the management of CAC focusing on several critical care issues: (1) laboratory testing for risk stratification and triage, (2) use of biomarkers to guide anticoagulation, (3) proposals for alterations of standard prophylactic VTE anticoagulation regimens for the prevention of thrombotic complications, (4) examination of available medications preferred for anticoagulation, (5) considerations for initiation of therapeutic anticoagulation, (6) indications for thrombolytic therapy, (7) decision-making regarding withholding anticoagulation treatment, (8) use of mechanical thromboprophylaxis, (9) monitoring of anticoagulation, (10) duration of therapeutic anticoagulation, (11) necessity of anticoagulation at discharge, and (12) treatment of active bleeding. We specifically focus on the recommendations and guidelines for critical care patients if mentioned in the major societal guidelines and recommendations; otherwise, we include the highest acuity patient population (i.e., hospitalized patients). A comparison of the similarities and differences between the guidelines and recommendations on each of the critical issues is provided.

**Table 1:** Major societal recommendations regarding laboratory testing for risk stratification and triage

Laboratory testing for risk stratification and triage	
**Centers for Disease Control and Prevention (CDC) guidelines** [6]	Mentions lack of prospective data demonstrating laboratory testing in risk stratification of patients who are asymptomatic or with mild infection. Mentions that there is insufficient data to recommend for or against using laboratory values to guide management.
**International Society for Thrombosis AND Haemostasis’s interim guidance (ISTH-IG)** [7]	Recommends obtaining D-dimer, PTT, platelet count, and fibrinogen levels for all patients who present with COVID-19 as the measurements may be helpful for risk stratification (D-dimer markedly raised three- to fourfold, prothrombin time prolonged, platelet count < 100 × 10^9^, and fibrinogen < 2.0 g/L) of patients who may need close monitoring and admission to hospital. Monitoring of parameters after admission may be helpful as more aggressive critical care treatment is warranted and experimental therapies should be considered if parameters worsen.
**Anticoagulation Forum (ACF)** [12]	Not mentioned
**American Society of Hematology (ASH)** [8, 9]	Recommends monitoring D-dimer, PTT, platelet count, and fibrinogen. Mentions that worsening of these parameters may predict more aggressive critical care and experimental therapies might be considered.
**American College of Chest Physicians (ACCP)** [10]	Not mentioned
**Scientific and Standardization Committee of ISTH (SCC-ISTH)** [11]	States further study is required. Acknowledges that use of very elevated D-dimer (> 6 times upper limit of normal) is a consistent predictor of thrombotic events and poor overall prognosis in this population.
**American College of Cardiology (ACC)** [13]	Similar to other acutely ill medical patients without COVID-19. Regular monitoring of platelet count, PT, D-dimer, and fibrinogen is important to diagnose worsening coagulopathy. Mentions that the treatment of underlying conditions of DIC and bacterial superinfections is important.

*Abbreviations*: *COVID-19* coronavirus disease 2019, *DIC* disseminated intravascular coagulation, *PT* prothrombin time, *PTT* partial thromboplastin time

**Table 2:** Major societal recommendations regarding using biomarkers to guide anticoagulation

Biomarkers to guide anticoagulation	
**CDC**	Insufficient data to recommend for or against using hematologic and coagulation parameters to guide management decisions.
**ISTH-IG**	Not mentioned
**ACF**	Biomarker thresholds such as D-dimer for guiding anticoagulation management should not be done outside the setting of a clinical trial.
**ASH**	No particular change to regimen recommended for patients with lupus like inhibitors. TEG and ROTEM should not be used routinely to guide management.
**ACCP**	Not mentioned
**SSC-ISTH**	D-dimer levels should not be used solely to guide anticoagulation regimens.
**ACC**	Further investigation is required to determine the role of antiphospholipid antibodies in pathophysiology of COVID-19-associated thrombosis. D-dimer > 2 times the upper limit may suggest that patient is at high risk for VTE and consideration of extended prophylaxis (up to 45 days) in patients at low risk of bleeding.

*Abbreviations*: *COVID-19* coronavirus disease 2019, *ROTEM* rotational thromboelastometry, *TEG* thromboelastography, *VTE* venous thromboembolism

**Table 3:** CDC and societal recommendations regarding thrombotic prophylaxis and treatment in COVID-19

	VTE prophylaxis regimen and preferred medications	Therapeutic anticoagulation regimens and preferred medications
**CDC**	LMWH or UFH (standard dosing). Insufficient data to recommend for or against the increase of anticoagulation intensity outside of a clinical trial.	Standard regimens for non-COVID-19 patients.
**ISTH-IG**	LMWH (standard dosing)	Not mentioned
**ACF**	Suggests an increased intensity of venous thromboprophylaxis be considered for critically ill patients^#^ (i.e., LMWH 40 mg SC twice daily, LMWH 0.5 mg/kg subcutaneous twice daily, heparin 7500 SC three times daily, or low-intensity heparin infusion) that they state is based largely on expert opinion.	LMWH over UFH whenever possible to avoid additional laboratory monitoring, exposure, and personal protective equipment. In patients with AKI or creatinine clearance < 15–30 mL/min, UFH is recommended over LMWH.
**ASH**	LMWH over UFH (standard dosing) to reduce exposure unless risk of bleeding outweighs risk of thrombosis.	LMWH or UFH over direct oral anticoagulants due to reduced drug-drug interactions and shorter half-life.
**ACCP**	LMWH (standard dosing)	LMWH or fondaparinux over UFH. UFH preferred in patients at high bleeding risk and in renal failure or needing imminent procedures. Recommend increasing dose of LMWH by 25–30% in patients with recurrent VTE despite therapeutic LMWH anticoagulation.
**SCC-ISTH**	LMWH or UFH. Intermediate intensity LMWH can be considered in high risk critically ill patients (50% of responders) and may be considered in non-critically ill hospitalized patients (30% of respondents). Mentions that there are several advantages of LMWH over UFH including once vs twice or more injections and less heparin-induced thrombocytopenia. Regimens may be modified based on extremes of body weight (50% increase in dose if obese), severe thrombocytopenia*, or worsening renal function.	Not mentioned
**ACC**	Enoxaparin 40 mg daily or similar LMWH regimen (i.e., dalteparin 5000 u daily) can be administered with consideration of SC heparin (5000 u twice to three times per day) in patients with renal dysfunction (i.e., creatinine clearance < 30 mL/min). Once daily regimens of LMWH may be advantageous over UFH to reduce missed doses associated with worse outcomes, reduce healthcare worker exposure, and conserve personal protective equipment. There is insufficient data to consider routine therapeutic or intermediate dose parenteral anticoagulation with UFH or LMWH. Only a minority of the panel considered intermediate intensity (31.6%; i.e., enoxaparin 1 mg/kg/day, enoxaparin 40 mg BID, UFH with target PTT 50–70) to therapeutic anticoagulation (5.2%) reasonable.	Medication regimen likely to change depending on comorbidities (i.e., renal or hepatic dysfunction, gastrointestinal function, thrombocytopenia). Parenteral anticoagulation (i.e., UFH) may be preferred in many ill patients given it may be withheld temporarily and has no known drug-drug interactions with COVID-19 therapies. LMWH may be preferred in patients who are unlikely to need procedures as there are concerns with UFH regarding the time to achieve therapeutic PTT and increased exposure to healthcare workers. DOACs have advantages including lack of monitoring that is ideal for outpatient management but may have risks in settings of organ dysfunction related to clinical deterioration and lack of timely reversal at some centers.

*Abbreviations*: *AKI* acute kidney injury, *BID* twice daily, *DOAC* direct oral anticoagulant, *i.e.* for example, *kg* kilograms, *LMWH* low molecular weight heparin, *mg* milligram, *min* minute, *mL* milliliter, *PTT* partial prothrombin time, *u* units, *SC* subcutaneous, *UFH* unfractionated heparin, *VTE* venous thromboembolism

*Platelet count not defined

^#^For non-critically ill hospitalized patients, standard dose thromboprophylaxis regimens are recommended

**Table 4:** CDC and societal recommendations regarding thrombotic prophylaxis and treatment in COVID-19

	When to hold anticoagulation	When to use mechanical thromboprophylaxis
**CDC**	Active hemorrhage or severe thrombocytopenia*	Not mentioned
**ISTH-IG**	Hold when signs of active bleeding or platelet count < 25 × 10^9^/L. Abnormal PT or PTT is not a contraindication to thromboprophylaxis.	Not mentioned
**ACF**	Active bleeding or profound thrombocytopenia*	Recommends intermittent pneumatic compression in patients with contraindication to pharmacological thromboprophylaxis. Mentions that it is reasonable to consider both mechanical and pharmacological thromboprophylaxis in critically ill patients if no contraindication exists for each modality.
**ASH**	Thromboprophylaxis is recommended even with abnormal coagulation tests in the absence of active bleeding and held only if platelet count < 25 × 10^9^/L or fibrinogen < 0.5 g/L. Abnormal PT or PTT is not a contraindication to thromboprophylaxis. Therapeutic anticoagulation may need to be held if platelet count < 30–50 × 10^9^/L or fibrinogen < 1.0 g/L.	Recommends mechanical thromboprophylaxis (i.e., pneumatic compression devices) when pharmacological thromboprophylaxis is contraindicated.
**ACCP**	Not mentioned	Suggest the use of mechanical thromboprophylaxis in critically ill patients who have a contraindication to pharmacological thromboprophylaxis. Suggest against the additional use of mechanical thromboprophylaxis in critically ill patients receiving pharmacological prophylaxis while mentioning that its addition is likely not to cause harm.
**SCC-ISTH**	No specific recommendations. Reports that 50% of respondents report holding if platelet count < 25 × 10^9^/L.	Mechanical thromboprophylaxis (intermittent pneumatic compression devices preferred) should be used when pharmacological therapy contraindicated. Multimodal thromboprophylaxis with mechanical methods (i.e., intermittent pneumonic compression devices) should be considered (60% of respondents).
**ACC**	In patients with moderate or severe COVID-19 on chronic therapeutic anticoagulation who develop suspected or confirmed DIC without overt bleeding, it is reasonable to consider the indication of anticoagulation and risk of bleeding for adjusting dose or discontinuation of anticoagulation. The majority of authors recommended reducing the intensity of anticoagulation unless there was an exceedingly high risk of thrombosis.	Mechanical thromboprophylaxis (intermittent pneumatic compression) should be considered in immobilized patients if pharmacological prophylaxis is contraindicated. Majority of panel members (55%) considered the use of both pharmacological thromboprophylaxis and intermittent pneumatic compression reasonable while acknowledging a lack of high-quality evidence.

*Abbreviations*: *COVID-19* coronavirus disease 2019, *i.e.* for example, *g* gram, *L* liter, *PT* prothrombin time, *PTT* partial thromboplastin time

*Platelet count not defined

**Table 5:** CDC and societal recommendations regarding therapeutic anticoagulation and thrombolytic therapy in COVID-19

	When to consider therapeutic anticoagulation	When to consider thrombolytics	Duration of therapeutic anticoagulation
**CDC**	Consider when a clinically suspected thromboembolic event is present or highly suspected despite imaging confirmation. Insufficient data to recommend for or against the increase of anticoagulation intensity outside the context of a clinical trial. Mentions patients who have thrombosis of catheters or extracorporeal filters should be treated accordingly to standard institutional protocols for patients without COVID-19.	Insufficient data to recommend for or against thrombolytic therapy outside the context of a clinical trial. In pregnant patients, thrombolytic therapy should only be used for acute PE with life-threatening hemodynamic instability due to risk for maternal hemorrhage.	Not mentioned
**ISTH-IG**	No specific recommendations	Not mentioned	Not mentioned
**ACF**	Consider when a clinically suspected thromboembolic event is present or highly suspected despite imaging confirmation.	Consider if clinical indication such as STEMI, acute ischemic stroke, or high risk massive PE with hemodynamic instability. Otherwise, it is not recommended outside context of a clinical trial.	A minimum of 3-month course for anyone who was initiated on anticoagulation during hospitalization (exception is recent bleeding or high bleeding risk). Delayed imaging should not be used to determine anticoagulation duration. Existing guidelines for presumed hospital-associated thromboembolic event should be used to determine anticoagulation beyond the initial 3-month period.
**ASH**	Consider increasing the intensity of anticoagulation regimen (i.e., from standard to intermediate intensity, from intermediate to therapeutic intensity) or change anticoagulants in patients who have recurrent thrombosis of catheters and extracorporeal circuits (i.e., ECMO, CRRT) on prophylactic anticoagulation regimens.	Not mentioned	Not mentioned
**ACCP**	Patients with PE or proximal DVT.	Thrombolytics over no such therapy in patients with objectively confirmed PE with hemodynamic instability or signs of obstructive shock who are not at high risk of bleeding. Peripheral thrombolysis recommended over catheter-directed thrombolysis.	Minimum of 3 months
**SCC-ISTH**	Therapeutic anticoagulation should not be considered for primary prevention until randomized controlled trials are available. Increased intensity of anticoagulation regimen (i.e., from standard or intermediate intensity to therapeutic intensity) can be considered in patients without confirmed VTE or PE but have deteriorating pulmonary status or ARDS.	Not mentioned	Minimum of 3 `months
**ACC**	Mentions that therapeutic anticoagulation is the key to VTE treatment. Does not make distinction between confirmed or suspected VTE. Hemodynamically stable patients with submassive PE should receive anticoagulation rather than fibrinolytics.	A multidisciplinary PERT may be helpful for intermediate and high risk patient with VTE. For hemodynamically high risk PE, systemic fibrinolysis is indicated with catheter-based therapies reserved for situations that are not amenable to systemic fibrinolysis. Patients with hemodynamically stable intermediate-low or intermediate-high risk PE should receive anticoagulation, and rescue systemic fibrinolysis should be considered in cases of further deterioration with catheter-directed therapies as an alternative. Catheter-directed therapies should be limited to most critical situations given minimal data showing mortality benefit. When considering fibrinolysis vs percutaneous coronary intervention for STEMI, clinicians should weigh risks and severity of STEMI presentation, severity of COVID-19 in patient, risk of COVID-19 to individual clinicians and healthcare system.	Not mentioned

*Abbreviations*: *ARDS* acute respiratory distress syndrome, *COVID-19* coronavirus disease 2019, *CRRT* continuous renal replacement therapy, *DVT* deep vein thrombosis, *ECMO* extracorporeal membrane oxygenation, *i.e.* for example, *PE* pulmonary embolism, *PERT* pulmonary embolism response team, *STEMI* ST elevation myocardial infarction, *VTE* venous thromboembolism

**Table 6:** CDC and societal recommendations regarding monitoring of anticoagulation in COVID-19

	Monitoring of patients receiving LMWH	Monitoring of patients with elevated PTT receiving therapeutic anticoagulation	Monitoring of patients receiving therapeutic anticoagulation
**CDC**	Not mentioned	Not mentioned	Per standard of care for patients without COVID-19
**ISTH-IG**	Advised in patients with severe renal impairment	Not mentioned	Not mentioned
**ACF**	Do not recommend dosing based on anti-Xa levels given lack of evidence on outcomes for thrombosis or bleeding	Recommend monitoring anti-Xa receiving UFH. LMWH use allows additional monitoring to be avoided.	Recommend monitoring anti-Xa levels to monitor UFH due to potential baseline PTT abnormalities. Reasonable to monitor anti-Xa or PTT in patients with normal baseline PTT levels and do not exhibit heparin resistance (> 35,000 u heparin over 24 h).
**ASH**	Not mentioned	May necessitate anti-Xa monitoring of UFH given artefactual increases in PTT.	May necessitate anti-Xa monitoring of UFH given artefactual increases in PTT.
**ACCP**	Body weight adjusted doses for LMWH do not require laboratory monitoring in majority of patients.	Not mentioned	Monitor anti-Xa levels in all patients receiving UFH given potential of heparin resistance.
**SCC-ISTH**	No specific recommendations. Mentions that LMWH may be advantageous over other agents for parenteral anticoagulation due to lack of routine monitoring.	Not mentioned	No specific recommendations. Mentions that expert clinical guidance statements and clinical pathways from large academic healthcare systems target an anti-factor Xa level of 0.3–0.7 IU/mL for UFH.
**ACC**	Not mentioned	Not mentioned	Not mentioned

*Abbreviations*: *COVID-19* coronavirus disease 2019, *IU* international unit, *LMWH* low molecular weight heparin, *mL* milliliter, *PTT* partial prothrombin time, *u* units

**Table 7:** CDC and societal recommendations regarding anticoagulation on discharge and correction of active bleeding in COVID-19

	Recommendations regarding anticoagulation on discharge	Correction of active bleeding
**CDC**	Routine venous thromboprophylaxis post-discharge is not recommended. FDA-approved prophylactic anticoagulation regimen (rivaroxaban and betrixaban) can be considered if high risk for VTE and low risk for bleeding using criteria from clinical trials.	Not mentioned
**ISTH-IG**	No specific recommendations	Transfuse to keep platelet count > 50 × 10^9^/L, fibrinogen > 1.5 g/L, PT ratio < 1.5
**ACF**	No evidence for anticoagulation beyond hospitalization, but reasonable to consider if low risk for bleeding and high risk for VTE including intubated, sedated, and paralyzed for multiple days.	Not mentioned
**ASH**	Reasonable to consider FDA-approved post-discharge prophylactic anticoagulation regimen (rivaroxaban and betrixaban) or aspirin if criteria from trials for post-discharge thromboprophylaxis are met.	Transfuse one adult unit of platelets if platelets < 50 × 10^9^/L, give 4 units of plasma if INR > 1.8, and fibrinogen concentrate (4 g) or cryoprecipitate (10 u) if fibrinogen < 1.5 g/L. In patients with severe coagulopathy and bleeding can consider 4F-PCC (25 u/kg) instead of plasma.
**ACCP**	Can be considered in patients who are at low risk of bleeding if emerging data suggests a clinical benefit.	Not mentioned
**SCC-ISTH**	Either LMWH or FDA-approved post-discharge prophylactic anticoagulation regimen (rivaroxaban and betrixaban) should be considered in patients with high VTE risk criteria. Duration is 14 days at least and up to 30 days. Of note, they report that none of the respondents recommended aspirin for post-discharge thromboprophylaxis.	Not mentioned
**ACC**	Reasonable to consider extended prophylaxis with LMWH or DOACs for up to 45 days in patients at high risk for VTE (i.e., D-dimer > 2 times the upper limit, reduced mobility, active cancer) and low risk of bleeding.	Transfuse platelets to maintain platelets > 50 × 10^9^/L in DIC and active bleeding or if platelets < 20 × 10^9^/L in patients at high risk of bleeding or requiring invasive procedures. FFP (15 to 25 mL/kg) in patients with active bleeding with either prolonged PT or PTT ratios (> 1.5 times normal) or decreased fibrinogen (< 1.5 g/L). Fibrinogen concentrate or cryoprecipitate in patients with persisting severe hypofibrinogenemia (< 1.5 g/L). Prothrombin complex concentrate if FFP is not possible. Tranexamic acid should not be used routinely in patients with COVID-19-associated DIC given the existing data.

*Abbreviations*: *COVID-19* coronavirus disease 2019, *DIC* disseminated intravascular coagulation, *DOAC* direct oral anticoagulant, *FDA* Food and Drug Administration, *FFP* fresh frozen plasma, *g* grams, *kg* kilograms, *PT* prothrombin time, *VTE* venous thromboembolism, *INR* international normalized ratio, *L* liter, *LMWH* low molecular weight heparin, *mL* milliliter, *PT* prothrombin time, *PTT* partial prothrombin time, *u* units, *4F-PCC* four-factor prothrombin complex concentrate

## Laboratory testing for risk stratification and triage

Both ISTH-IG and ASH are the only societies that recommend monitoring D-dimer, PTT, platelet count, and fibrinogen levels for risk stratification of critical care treatment and consideration of experimental COVID-19 therapies based on changes in these parameters. ISTH-IG also recommends obtaining D-dimer, PTT, platelet count, and fibrinogen for all patients who present with COVID-19 infection to help guide which patients may require admission. Specifically, they provide an algorithm and the following thresholds for admission to the hospital: D-dimer markedly raised three- to fourfold, prothrombin time prolonged, platelet count < 100 × 10^9^, and fibrinogen < 2.0 g/L. Alternatively, the CDC states that there is a lack of prospective data demonstrating laboratory measures of coagulopathy for risk stratification in patients who are asymptomatic or with mild infection, and insufficient data to recommend for or against using such laboratory values to guide management. The SCC-ISTH mentions that the risk stratification algorithm for hospitalized patients requires further study while acknowledging the use of very elevated D-dimer (> 6 times upper limit of normal (ULN)) appears to be a consistent predictor of thrombotic events and poor overall prognosis in this patient population. ACC recommends that risk stratification of COVID-19 patients should be similar to other acutely ill medical patients without COVID-19 while stating that regular monitoring of platelet count, PT, D-dimer, and fibrinogen is important to diagnosing worsening coagulopathy and treatment of underlying conditions of DIC and bacterial superinfections is important.

## Using biomarkers to guide intensity of anticoagulation

None of the societies recommend daily monitoring of biomarkers as a means to guide intensity of anticoagulation management. The ACF states that biomarker thresholds such as D-dimer for guiding anticoagulation management should not be done outside the setting of a clinical trial. Similarly, the SCC-ISTH remarks that D-dimer levels should not be used to solely to guide anticoagulation regimens. Only ASH and ACC address the issue of anticoagulation in patients with lupus anticoagulant inhibitors. ASH recommends that those patients be treated with prophylactic anticoagulation regimens similar to other hospitalized patients, and ACC mentions that further investigation is required to determine the role of antiphospholipid antibodies in pathophysiology of CAC. ASH also mentions that thromboelastography (TEG) and rotational thromboelastometry (ROTEM) are currently under investigation for CAC and disseminated intravascular coagulopathy (DIC) and should not be used routinely to guide management. The ACC mentions that D-dimer > 2 times ULN may suggest that patient is at high risk for VTE and to consider extended prophylaxis (up to 45 days) in patients with low risk of bleeding.

## Alterations from standard venous thromboembolism anticoagulation regimens to prevent thrombotic complications

None of the major societies recommend therapeutic anticoagulation for prevention of thrombotic complications. Only the ACF recommends increasing the intensity of anticoagulation in critically ill patients (enoxaparin 40 mg twice daily or heparin 7500 unit/dose three times daily) while they recommend standard dose anticoagulant prophylaxis for all other hospitalized non-critically ill patients. The SCC-ISTH mentions that intermediate LMWH dosing can be considered in high risk critically ill patients (50% of respondents) and may be considered in non-critically ill hospitalized patients (30% of respondents). The SCC-ISTH also specifically mentions that the anticoagulation regimens may be modified based on extremes of body weight (50% increase in dose if obese), severe thrombocytopenia, or worsening renal function. The ACC mentions that while there is insufficient data to consider routine intermediate or therapeutic dosing, a minority of their panel considers an intermediate intensity (31.6%; i.e., enoxaparin 1 mg/kg/day, enoxaparin 40 mg BID, UFH with target PTT 50–70) or therapeutic anticoagulation (5.2%) reasonable. The CDC recommends that any changes in anticoagulation regimens that are different than the current standard of care for patients without COVID-19 should only be administered in the setting of a clinical trial.

## Preferred medication regimens for prophylactic anticoagulation

Many of the societies recommend using once daily prophylactic anticoagulants to reduce healthcare worker exposure. The ISTH-IG, ASH, and ACCP recommend using LMWH in critically ill patients. ASH particularly mentions that LMWH is preferred over UFH to reduce exposure unless the risk of bleeding outweighs the risk of thrombosis. The CDC, ACF, and SCC-ISTH recommend LMWH or UFH with the rationale provided by the CDC being due to their short half-lives, versatility in administration (IV or subcutaneously), and less drug-drug interactions compared to oral anticoagulants. The SCC does particularly mention that there are several advantages of LMWH over UFH including once vs twice or more injections and less heparin-induced thrombocytopenia. Similarly, the ACC mentions that enoxaparin 40 mg daily or similar LMWH regimen (i.e., dalteparin 5000 u daily) can be administered with consideration of subcutaneous heparin (5000 u twice to three times per day) in patients with renal dysfunction (i.e., creatinine clearance < 30 mL/min). They mention that once daily regimens of LMWH may be advantageous over UFH to reduce missed doses associated with worse outcomes, reduce healthcare worker exposure, and conserve personal protective equipment. None of the societies recommend routine use of direct oral anticoagulants because of potential drug-drug interactions, renal and hepatic function abnormalities that have implications on pharmacodynamics, longer half-lives, cost implications, and/or lack of reversal agents in some hospitals (specifically mentioned in CDC, SCC-ISTH, Anticoagulation Forum, and ACCP guidelines).

## Preferred medications regimens for therapeutic anticoagulation

The ACF and ACCP generally prefer LMWH over UFH to decreases staff exposure and laboratory monitoring. The ACCP also prefers fondaparinux over UFH and mentions that UFH may be preferred in patients at high bleeding risk, with renal failure or needing imminent procedures. Similarly, the ACF recommends UFH over LMWH in patients with AKI or creatinine clearance < 15–30 mL/min. ASH mentions that LMWH or UFH is preferred over oral anticoagulants due to potential drug-drug interactions and their shorter half-lives. The SCC-ISTH and ISTH-IG do not mention preferred medication regimens for therapeutic anticoagulation. The CDC mentions that standard regimens for patients without COVID-19 patients should be used. The ACC mentions that the medication regimen is likely to change depending on comorbidities (i.e., renal or hepatic dysfunction, gastrointestinal function, thrombocytopenia). They give no particular preference to an agent but do mention that (1) parenteral anticoagulation (i.e., UFH) may be preferred in many ill patients given it may be withheld temporarily and has no known drug-drug interactions with COVID-19 therapies, (2) LMWH may be preferred in patients who are unlikely to need procedures as there are concerns with UFH regarding the time to achieve therapeutic PTT and increased exposure to healthcare workers, and (3) direct oral anticoagulants (DOAC) have advantages including lack of monitoring that are ideal for outpatient management but have risks in settings of organ dysfunction related to clinical deterioration and lack of timely reversal at some centers.

## Withholding anticoagulation

Most of the major societal guidelines and recommendations (ISTH-IG, ACF, CDC, and ASH) advise holding anticoagulation in patients who are actively bleeding or severely thrombocytopenic. ISTH-IG recommends holding LMWH prophylactic anticoagulation for patients with platelet count less than 25 × 10^9^/L. ASH mentions that therapeutic anticoagulation should be held if platelet count < 30–50 × 10^9^/L or fibrinogen < 1.0 g/L, and prophylactic anticoagulation should be held only if platelet count < 25 × 10^9^/L or fibrinogen < 0.5 g/L. The SCC-ISTH does not recommend a particular platelet level to hold anticoagulation but does report that 50% of their respondents report holding for a platelet count < 25 × 10^9^/L. While the CDC and ACF recommend holding prophylactic anticoagulation in patients who are severely thrombocytopenic, they also do not recommend a particular platelet count threshold. The ACCP does not make any specific recommendations regarding the holding of anticoagulation. The ACC mentions that in patients with moderate or severe COVID-19 on chronic therapeutic anticoagulation who develop suspected or confirmed DIC with overt bleeding, it is reasonable to consider the indication of anticoagulation and risk of bleeding for adjusting dose or discontinuation of anticoagulation. The majority of their authors recommend reducing the intensity of anticoagulation unless there is an exceedingly high risk of thrombosis. Of note, the ISTH-IG, ACF, and ASH mention that abnormal PT or PTT is not a contraindication to thromboprophylaxis.

## Use of mechanical thromboprophylaxis

The ACF, ASH, ACCP, ACC, and SCC-ISTH recommend or suggest mechanical thromboprophylaxis when pharmacological thromboprophylaxis is contraindicated. The ACF, ASH, ACC, and SCC-ISTH particularly mention pneumatic compression devices as examples of mechanical thromboprophylaxis. The ACF mentions that it is reasonable to consider both mechanical and pharmacological thromboprophylaxis in critically ill patients if no contraindication exists for each modality. The SCC-ISTH mentions that multimodal thromboprophylaxis with mechanical methods (i.e., intermittent pneumonic compression devices) should be considered (60% of respondents). Similarly, the majority of ACC panel members (55%) considered the use of both pharmacological thromboprophylaxis and intermittent pneumatic compression reasonable while acknowledging a lack of high-quality evidence. The ACCP suggests against the additional use of mechanical thromboprophylaxis in critically ill patients receiving pharmacological prophylaxis while mentioning that its addition is likely not to cause harm. The CDC and ISTH-IG do not mention any recommendations or suggestions regarding the use of mechanical thromboprophylaxis.

## Considerations for therapeutic anticoagulation

The CDC, ACCP, and ACF do not recommend therapeutic anticoagulation except in cases where there is a thromboembolic event or a high suspicion for a thromboembolic event when imaging is not possible. ASH recommends therapeutic anticoagulation only if there is a documented clinical indication (e.g., VTE, atrial fibrillation, or mechanical valve). ACC mentions that therapeutic anticoagulation is the key to VTE treatment but does not make a distinction between confirmed or suspected VTE. They do mention that hemodynamically stable patients with intermediate-low to intermediate-high risk PE should be managed initially with anticoagulation and close monitoring rather than fibrinolytics. While the SCC-ISTH recommends that therapeutic anticoagulation should not be considered for primary prevention until randomized trials are available, they do mention that therapeutic anticoagulation (i.e., changing from standard or intermediate intensity to therapeutic intensity) can be considered in patients without confirmed VTE but who have deteriorating pulmonary or ARDS. ASH also states that it is reasonable to consider increasing the intensity of the patient’s anticoagulation regimen (i.e., from standard to intermediate intensity, from intermediate to therapeutic intensity) or change anticoagulants in patients who have recurrent thrombosis of catheters and extracorporeal circuits (i.e., ECMO, CRRT) on prophylactic anticoagulation regimens. Similarly, the ACCP recommends increasing the dose of LMWH by 25–30% in patients with recurrent VTE despite being on therapeutic LMWH anticoagulation. The CDC specifically mentions that patients who have thrombosis of catheters or extracorporeal filters should be treated accordingly to standard institutional protocols for patients without COVID-19.

## Considerations for thrombolytic treatment?

The ACF recommends against the use of thrombolytics outside of a clinical trial unless there is a clinical indication for which it is already approved such as ST elevation myocardial infarction, acute ischemic stroke, or massive PE with hemodynamic instability. The CDC also mentions that there is insufficient data to recommend for or against thrombolytic therapy outside the context of a clinical trial. However, the CDC specifically mentions that in pregnant patients in particular, thrombolytic therapy should only be used for acute PE with life-threatening hemodynamic instability due to risk for maternal hemorrhage. The ISTH-IG, ASH, and SCC-ISTH do not mention any recommendations or suggestions regarding the use of thrombolytic treatment.

The ACCP suggests the use of thrombolytic therapy in patients with massive PE (i.e., hypotension with systolic blood pressure < 90 mmHg or when there are signs of obstructive shock) and specifically recommends peripheral thrombolysis using a peripheral vein over catheter-directed thrombolysis. In addition, ACCP suggests that in patients in whom therapeutic anticoagulation fails and who continue to have evidence of cardiopulmonary compromise, thrombolytic therapy may be beneficial. Similarly, the ACC mentions that systemic fibrinolysis is indicated in patients with significant hemodynamically unstable high risk PE and catheter-based therapies be reserved for situations that are not amenable to systemic fibrinolysis. Furthermore, hemodynamically stable patients with intermediate-low or intermediate-high risk PE should receive anticoagulation and fibrinolysis or catheter-directed therapies should be considered in cases of further deterioration. They also report that a multidisciplinary PE response team (PERT) may be helpful for intermediate and high risk patients with VTE and that catheter-directed therapies should be limited to most critical situations given minimal data demonstrating a mortality benefit.

## Duration of therapeutic anticoagulation

The ACF recommends at least a 3-month course of anticoagulation for patients who are started on anticoagulation for a presumed provoked thrombus from the inflammatory state of CAC but did not have imaging available for confirmation. The ACF also recommends that standard anticoagulation guidelines be used to determine length of anticoagulation beyond the initial 3-month period. The ACF mentions that an exception to the minimum 3-month course is in patients with recent bleeding or high risk of bleeding. In addition, the ACF comments that if confirmatory imaging cannot be obtained within a couple days of presumed diagnosis, it is possible that delayed imaging may show an absence of thrombus when one was present initially. Therefore, they recommend continuing empiric treatment for the minimum 3-month course. Similarly, the ACCP and SCC-ISTH recommend a minimum of 3 months of anticoagulation in those patients with confirmed PE or proximal DVT. The ISTH-IG, ASH, ACC, and CDC do not mention any recommendations or suggestions regarding the duration of therapeutic anticoagulation.

## Monitoring of anticoagulation

### Monitoring of patients receiving LMWH

None of the guidelines recommend or mentions routine monitoring for LMWH anticoagulation, with the exception of ISTH-IG which advises monitoring in patients with severe renal impairment receiving LMWH prophylactic anticoagulation. The ACF does not recommend dosing LMWH based on anti-Xa levels, and ACCP mentions that LMWH can be administered without laboratory monitoring in the majority of patients. The CDC, ACC, and ASH do not have particular monitoring recommendations other than the current standard of care for other hospitalized patients.

### Monitoring of patients with prolonged PTT levels receiving therapeutic anticoagulation

The ACF is the only society to recommend monitoring anti-Xa levels in patients with prolonged baseline PTT levels receiving therapeutic anticoagulation with UFH. While both ASH and ACCP do not make any specific recommendations regarding patients with prolonged PTT levels receiving therapeutic anticoagulation with UFH, ASH does mention that monitoring anti-Xa levels for patients receiving UFH may be necessary given artefactual increases in PTT and ACCP recommends monitoring anti-Xa levels in all patients receiving UFH given a potential for heparin resistance. The ISTH-IG, CDC, ACCP, ACC, and SCC-ISTH do not mention any recommendations or suggestions regarding monitoring of patients with prolonged PTT levels receiving therapeutic anticoagulation.

### Monitoring of patients receiving therapeutic anticoagulation

None of the societal guidelines and recommendations make specific monitoring recommendations for patients receiving therapeutic anticoagulation with the exception of the ACF and ACCP which both recommend monitoring anti-Xa levels to monitor UFH due to baseline abnormalities in PTT. ACF does mention that it is reasonable to monitor anti-Xa or PTT in patients without prolonged PTT at baseline receiving UFH for therapeutic anticoagulation. ASH particularly mentions that anti-Xa monitoring of UFH may be necessary given artefactual increases in PTT secondary to lupus-like inhibitors. The SCC-ISTH does not make any specific recommendations regarding the use of anti-Xa levels to monitor UFH but does mention that expert clinical guidance statements and clinical pathways from large academic healthcare systems target an anti-factor Xa level of 0.3–0.7 IU/mL for UFH.

## Necessity of anticoagulation at discharge

The CDC and ACF recommend against the routine use of prophylactic anticoagulation for patients discharged from the hospital. However, the CDC and ACF as well as ASH and SCC-ISTH mention that FDA-approved post-discharge prophylactic anticoagulation (PDPA) regimens (rivaroxaban and betrixaban) may be considered in patients with high risk for VTE and low risk for bleeding. Similarly, the ACCP mentions that PDPA can be considered in patients who are at low risk of bleeding if emerging data suggests that the risk of VTE outweighs the risk of bleeding. The ACF and SCC-ISTH both mention that enoxaparin in addition to betrixaban or rivaroxaban can be considered if PDPA is thought to be reasonable. The duration of anticoagulation recommended by ACF is based on the timing used in clinical trials which is 31–39 days for rivaroxaban, 35–42 days for betrixaban, and 6–14 days for enoxaparin. The SCC-ISTH recommends a duration of a minimum of 14 days and up to 30 days. ACC mentions that it is reasonable to consider extended prophylaxis with LMWH or DOACs for up to 45 days in patients at high risk for VTE (i.e., D-dimer > 2 times ULN, reduced mobility, active cancer) and low risk of bleeding. As an alternative, ASH mentions that aspirin can also be considered based on studies for VTE prophylaxis in low risk patients after orthopedic surgery. Aspirin is not mentioned in any of the other guidelines and recommendations with the exception of SCC-ISTH which reports that none of their respondents recommended aspirin for post-discharge thromboprophylaxis.

## Correction of active bleeding

In the setting of bleeding, the ISTH-IG recommends transfusion to keep platelet count > 50 × 10^9^/L, fibrinogen > 1.5 g/L, and PT ratio < 1.5. ACC recommends platelet transfusion to maintain platelet count > 50 × 10^9^/L in DIC and active bleeding (> 20 × 10^9^/L in patients at high risk of bleeding or requiring invasive procedures), fresh frozen plasma (FFP) (15 to 25 mL/kg) in patients with active bleeding with either prolonged PT or PTT ratios (> 1.5 times normal) or decreased fibrinogen (< 1.5 g/L), and fibrinogen concentrate or cryoprecipitate in patients with persisting severe hypofibrinogenemia (< 1.5 g/L). Similarly, ASH recommends transfusion if platelets < 50 × 10^9^/L, INR < 1.8, and fibrinogen < 1.5 g/L. ASH also recommends that in the setting of severe coagulopathy and bleeding, 4F-PCC be considered instead of plasma so as to prevent significant volume overload. ACC mentions that prothrombin complex concentrate is recommended if FFP is not possible and tranexamic acid should not be used routinely in patients with COVID-19-associated DIC given the existing data. Moreover, ASH mentions that transfusions to simply to correct laboratory abnormalities in the absence of bleeding may worsen disseminated thrombosis and deplete blood product resources without any evidence of improving clinical outcomes. Similarly, ACC mentions that correction of coagulopathy in unselected patients without significant bleeding is not recommended.

## Implications for clinical practice and future investigation

These societal guidelines are based on consensus of expert opinion with limited evidence available in the form of robust clinical trials. Consequently, it is not surprising there are clinical issues that are either not addressed by the guidelines or have varying opinions. For instance, practitioners who deem a critically ill COVID-19 patient at high risk for thrombotic complication (e.g., trauma, cancer, antiphospholipid antibody) may decide to use a higher intensity prophylactic anticoagulation regimen consistent with the ACF guidelines rather than routine anticoagulation prophylactic regimen recommended by other societies. The major societal guidelines and recommendations share significant similarities, but also have some discrepancies. We recommend that providers manage their patients in the framework of these major societal guidelines, and where discrepancies do exist, decisions be made based on the practitioner’s experience and their understanding of a patient’s medical history, clinical course, and perceived risk.

Based on this review and comparison of the societal guidelines, there are a number of unaddressed issues that warrant further investigation and incorporation into updated guidelines. Further studies of COVID-19 pathophysiology are required to elucidate the mechanisms of coagulation disruption in order to identify potential pharmacologic targets and new biomarkers for risk stratification. Large randomized control trials are needed to investigate the optimal prophylactic anticoagulation regimen, the utility of intermediate and therapeutic anticoagulation, and the use of fibrinolytic therapy in COVID-19 patients. There are several clinical trials currently in the ClinicalTrials.gov database focused on optimizing prophylactic anticoagulation regimens (8 total) (Table 8), therapeutic anticoagulation (5 total) (Table 9), and fibrinolytic therapy (2 total) (Table 10) in COVID-19 patients [14], and we eagerly await those results. Further guidance from prospective studies is required to guide the clinical significance of biomarkers (e.g., D-dimer, antiphospholipid, and lupus anticoagulant antibodies) in risk stratification and treatment of CAC.

**Table 8:** Ongoing clinical trials optimizing prophylactic anticoagulation regimens in COVID-19

NCT number	Study name	Interventions	Sponsor
NCT04409834	Prevention of Arteriovenous Thrombotic Events in Critically-Ill COVID-19 Patients Trial	Evaluation of varying anticoagulation strategies including UFH IV, UFH SC, enoxaparin 1 mg/kg, enoxaparin 40 mg SC, and clopidogrel	The TIMI Study Group
NCT04367831	Intermediate or Prophylactic- Dose Anticoagulation for Venous or Arterial Thromboembolism in Severe COVID-19	Evaluation of prophylactic enoxaparin vs intermediate dosing	Columbia University (USA)
NCT04367831	Patient Characteristics, Outcome and Thromboembolic Events Among Adult Critically Ill COVID-19 Patients with Different Anticoagulant Regimes	Completed dose finding study of tinzaparin	Karolinska Institute (Sweden)
NCT04367831	Preventing COVID-19 Complications with Low- and High-dose Anticoagulation	Evaluating low vs high dose enoxaparin	University Hospital Geneva (Switzerland)
NCT04344756	Trial Evaluating Efficacy and Safety of Anticoagulation in Patients With COVID-19 Infection, Nested in the Corimmuno-19 Cohort	Evaluation of tinzaparin vs UFH anticoagulation	Assistance Publique – Hȏpitaux de Paris (France)
NCT04360824	Covid-19 Associated Coagulopathy	Evaluation of prophylactic vs intermediate dose thromboprophylaxis	University of Iowa (USA)
NCT04360824	Full Dose Heparin Vs. Prophylactic or Intermediate Dose Heparin in High Risk COVID-19 Patients	Evaluation of prophylactic vs intermediate dose thromboprophylaxis	Northwell Health (USA)
NCT04372589	Full Dose Heparin Vs. Prophylactic or Intermediate Dose Heparin in High Risk COVID-19 Patients	Evaluation of heparin dose strategy	University of Manitoba (Canada)

*Abbreviations*: *COVID-19* coronavirus disease 2019, *SC* subcutaneous, *UFH* unfractionated heparin

**Table 9:** Ongoing clinical trials of therapeutic anticoagulation in COVID-19

NCT number	Study name	Interventions	Sponsor
NCT04377997	Safety and Efficacy of Therapeutic Anticoagulation on Clinical Outcomes in Hospitalized Patients With COVID-19	Evaluation of therapeutic enoxaparin	Massachusetts General Hospital (USA)
NCT04394377	Full Anticoagulation Versus Prophylaxis in COVID-19: COALIZAO ACTION Trial	Evaluation of therapeutic anticoagulation with rivaroxaban 20 mg/day vs prophylactic enoxaparin 40 mg/day	Brazilian Clinical Research Institute (Brazil)
NCT04359277	A Randomized Trial of Anticoagulation Strategies in COVID-19	Evaluation of therapeutic high dose enoxaparin vs standard prophylactic dose	NYU Langone Health (USA)
NCT04359277	Evaluation of therapeutic high dose Enoxaparin versus standard prophylactic dose Anticoagulation in Critically Ill Patients With COVID-19 (The IMPACT Trial)	Evaluation of varying anticoagulation regimens including enoxaparin, UFH, fondaparinux, and argatroban	Weill Medical College of Cornell University (USA)
NCT04362085	Coagulopathy of COVID-19: A Pragmatic Randomized Controlled Trial of Therapeutic Anticoagulation Versus Standard Care	Evaluation of therapeutic anticoagulation vs standard of care prophylaxis	St Michael’s Hospital (Canada)

*Abbreviations*: *COVID-19* coronavirus disease 2019, *UFH* unfractionated heparin

**Table 10:** Ongoing clinical trials of fibrinolytic therapy in COVID-19

NCT number	Study name	Interventions	Sponsor
NCT04357730	Fibrinolytic Therapy to Treat ARDS in the Setting of COVID-19 Infection	Phase 2a trial comparing 50 mg and 100 mg doses of alteplase	Denver Health and Hospital Authority (USA)
NCT04356833	Nebulised rtPA for ARDS Due to COVID-19 (PACA)	Phase 2 trial evaluation nebulized tissue plasminogen activator in COVID-19 ARDS	University College London (UK)

*Abbreviations*: *ARDS* acute respiratory distress syndrome, *COVID-19* coronavirus disease 2019, *mg* milligrams, *Rt-PA* recombinant tissue plasminogen activator

## Conclusions

There are significant similarities and differences surrounding the major societal recommendations and guidelines regarding risk stratification and management of COVID-19-associated coagulopathy in regard to use of laboratory makers, prevention, treatment, and monitoring of VTE, and post-discharge thromboprophylaxis. In addition, there are a number of unanswered questions that warrant further investigation. It is incumbent on the clinicians to remain updated on emerging literature in this area in order to optimize the care of critically ill patients with COVID-19.

## Data Availability

Not applicable.

## Abbreviations

ACF: Anticoagulation Forum
ACC: American College of Cardiology
ACCP: American College of Chest Physicians
ASH: American Society of Hematology
ARDS: Acute respiratory distress syndrome
CAC: COVID-19-associated coagulopathy
CDC: Centers for Disease Control and Prevention
COVID-19: Coronavirus disease 2019
CRRT: Continuous renal replacement therapy
DVT: Deep venous thrombosis
DIC: Disseminated intravascular coagulation
ECMO: Extracorporeal membrane oxygenation
ISTH: International Society on Thrombosis and Haemostasis
LMWH: Low molecular weight heparin
PT: Prothrombin time
PTT: Partial thromboplastin time
PE: Pulmonary embolism
SCC: Scientific and Standardization Committee
STEMI: ST elevation myocardial infarction
UFH: Unfractionated heparin
ULM: Upper limit of normal
